# Drug repurposing: insights into the antimicrobial effects of AKBA against MRSA

**DOI:** 10.1186/s13568-024-01660-0

**Published:** 2024-01-06

**Authors:** Yingjia Li, Hongbing Ma

**Affiliations:** The Second Affiliated Hospital of Xi’an Jiaotong University, Xi’an, 710004 China

**Keywords:** *Staphylococcus aureus*, Biofilm, Persister cells, AKBA, Drug repurposing

## Abstract

*Staphylococcus aureus* is a major threat in infectious diseases due to its varied infection types and increased resistance. *S. aureus* could form persister cells under certain condition and could also attach on medical apparatus to form biofilms, which exhibited extremely high resistance to antibiotics. 3-Acetyl-11-keto-beta-boswellic acid (AKBA) is a well-studied anti-tumor and antioxidant drug. This study is aimed to determine the antimicrobial effects of AKBA against *S. aureus* and its persister cells and biofilms. The in vitro antimicrobial susceptibility of AKBA was assessed by micro-dilution assay, disc diffusion assay and time-killing assay. Drug combination between AKBA and conventional antibiotics was detected by checkerboard assay. And the antibiofilm effects of AKBA against *S. aureus* were explored by crystal violet staining combined with SYTO/PI probes staining. Next, RBC lysis activity and CCK-8 kit were used to determine the cytotoxicity of AKBA. In addition, murine subcutaneous abscess model was used to assess the antimicrobial effects of AKBA in vivo. Our results revealed that AKBA was found to show effective antimicrobial activity against methicillin-resistant *S. aureus* (MRSA) with the minimal inhibitory concentration of 4–8 µg/mL with undetectable cytotoxicity. And no resistant mutation was induced by AKBA after 20 days of consecutive passage. Further, we found that AKBA could be synergy with gentamycin or amikacin against *S. aureus* and its clinical isolates. By crystal violet and SYTO9/PI staining, AKBA exhibited strong biofilm inhibitory and eradication effects at the concentration of 1 ~ 4 µg/mL. In addition, the effective antimicrobial effect was verified in vivo in a mouse model. And no detectable in vivo toxicity was found. These results indicated that AKBA has great potential to development as an alternative treatment for the refractory *S. aureus* infections.

**Supplementary Information:**

The online version contains supplementary material available at 10.1186/s13568-024-01660-0.

## Introduction

*Staphylococcus aureus* (*S. aureus*) is a common Gram-positive pathogen that often causes skin and soft tissue infections, respiratory infections, osteomyelitis, and medical device implant related infections in patients with immunodeficiency. In severe cases, it can lead to lethal blood infections such as sepsis (Vestergaard et al. 2019; Cheung et al. 2021). With the abuse of antibiotics, the resistance rate of *S. aureus* continues to increase, and the emergence of methicillin-resistant *S. aureus* (MRSA) has become one of the major challenges in clinical settings (Hou et al. 2023).

In recent years, researchers have discovered a highly resistant phenotype of *S. aureus*, namely persister cells (Kim et al. 2019). The high drug resistance of the persister cells is caused by a phenotypic change. When the bacteria are in a plateau period of growth, they form persister cells, due to nutrient deficiency or other adverse environmental factors, by activating stress response mediated by the second messenger (p)ppGpp (Prax and Bertram 2014; Chang et al. 2020). Due to the non-proliferation and inactive metabolism of the persister cells, as well as the inhibition of ATP production, their drug resistance is greatly increased. Persister cells formed by *S. aureus* are resistant to almost all commoditized antibiotics, and are one of the main reasons for the persistence of refractory infections (Chang et al. 2020). Therefore, developing new antibacterials targeting MRSA and its persister cells has become an urgent problem to be solved.

*S. aureus* can adhere to the surface of medical implants and further aggregate to form a biofilm (Lister and Horswill 2014). The extracellular matrix of the biofilm can effectively prevent the penetration of antibiotics, thereby greatly increased the bacterial resistance. The use of conventional antibiotics alone often makes it difficult to effectively eradicate the biofilm that has already pre-formed, posing great difficulties for clinical treatment (Guo et al. 2022). Therefore, studying antibacterial drugs targeting mutidrug-resistant *S. aureus* and its biofilms is of great clinical significance.

Drug repurposing, which refers to the reuse of well-studied drugs apart from its original targets, has been a research hotspot in recent years. It has many irreplaceable advantages over traditional drug development. Most of these drugs have clear targets, pharmacokinetic and toxicological data, which greatly save research time and costs (Zheng et al. 2018). And drug repurposing is particularly suitable for the development of drugs for explosive epidemics. For example, in recent years, by drug repurposing, researchers found that the well marketed drugs, such as radcivir, favepiravir, ribavirin, lobinavir, litonavir, darunavir, abido, chloroquine and hydroxychloroquine, have significant antiviral effects against coronavirus (Singh et al. 2020).

3-Acetyl-11-keto-beta-boswellic acid (AKBA), a pentacyclic triterpenoid, is a well-studied anti-tumor as well as antioxidant reagent (Ding et al. 2016; Kumar et al. 2023). And it also shows biological activities in nervous system diseases (Gong et al. 2022). However, to the best of our knowledge, there is no study reported the antimicrobial effects of AKBA against *S. aureus*. Thus, in our present study, we will explore the in vitro and in vivo antimicrobial activities of AKBA, and further assess its effects against *S. aureus* high resistant phenotypes, for example, biofilms and persister cells.

## Materials and methods

### Bacterial strains and culture conditions

The MRSA strains (including ATCC 43,300 and USA300), MSSA strains (including ATCC 25,923 and MW2) and *Staphylococcus epidermidis* RP62A and ATCC 12,228 were purchased form American Type Culture Collection or BNCC BeNa Culture Collection (Beijing, China). Other type strains of *Escherichia coli* ATCC 25,923, *Enterococcus faecalis* ATCC 29,212, *Klebsiella pneumoniae* ATCC 700,603, *Acinetobacter baumannii* ATCC 19,606 and *Pseudomonas aureginosa* PAO1 were provided by Luo Juncai (Tiandiren Biotech, Changsha, China). Clinical strains of MRSA were isolated from the Third Xiangya Hospital of Central South University, and identified by Matrix-Assisted Laser Desorption Ionization (BD, Germany). *S. aureus* and *S. epidermidis* were grown in Tryptic Soy Broth (TSB), *E. faecalis* was grown in Brain Heart Infusion (BHI). Other Gram-negative strains were grown in Luria-Bertani (LB) broth. All the culture mediums were purchased form Solarbio (Beijing, China). Chemicals of AKBA and other antibiotics were purchased from the MedChem Express (New Jersey, USA) and dissolved in dimethyl sulfoxide (DMSO) or deionized water.

### Micro-dilution assay for antimicrobial susceptibility determination

The antimicrobial susceptibility of antimicrobials against pathogens was determined according to the recommendation of the Clinical and Laboratory Standards Institute (CLSI) (CLSI 2023). Briefly, single colony of overnight cultured *S. aureus* was adjusted to 0.5 McFarland (McF) in Mueller-Hinton (MH) II broth. Then, the bacterial suspension was 1: 100 diluted with MH borth, and mixed with equal volumes of 2-fold diluted antimicrobials. After incubation at 37℃ for 16-18 h, the minimal inhibitory concentration (MIC) was determined as the lowest concentration with no visible bacterial growth. Further, an aliquot of supernatant form 1× MIC to the uppermost concentration was removed on a MH agar. After overnight incubation, the minimal bactericidal concentration (MBC) was determined as the lowest concentration with no visible colony formation.

### Disc diffusion assay

The procedure was designed in accordance with the recommendation of the CLSI (CLSI 2023). The log-phased *S. aureus* ATCC 43,300 was adjusted to 0.5 McF with saline, and the bacterial suspension was spread onto the MH agar plate with a sterile swab. The commercial blank disks (diameter = 6 mm) containing DMSO or AKBA were loaded on the surface. After incubating for 16–18 h at 37 ℃, the inhibitory zone was measured by a caliper.

### Time-killing assay

*S. aureus* was cultured to Log-phased growth and adjusted to ~ 1 × 10^6^ CFU/mL in MH broth in centrifuge tubes. Equal volumes of 2-fold MH broth diluted antimicrobials were added to each tube. The bacterial suspension was then incubated at 37 ℃ at 180 rpm. An aliquot of the suspension was removed to sheep blood agar to perform CFU counting at the time point of 0, 2, 4, 8, 12 and 24 h, respectively (Liu Y. 2021).

### Drug combination determination

The antimicrobial interactions between AKBA and conventional antibiotics were evaluated by checkerboard assay (Mahomoodally et al. 2018). Firstly, the MICs of antimicrobials to be tested were determined as described above. Then, 2-fold dilutions of two drugs were mixed horizontally and vertically, respectively, at equal volumes in a 96-well plate in the presence of ~ 1 × 10^6^ CFU/mL *S. aureus*. After incubated at 37 ℃ for 16–18 h, the OD_630nm_ was obtained, and the calculation formula for the fractional inhibition concentration index (FICI) is as follows:

$$FICI=\frac{{MIC}_{\text{A} \text{i}\text{n} \text{c}\text{o}\text{m}\text{b}\text{i}\text{n}\text{a}\text{t}\text{i}\text{o}\text{n}}}{{MIC}_{\text{A} \text{a}\text{l}\text{o}\text{n}\text{e}}}+\frac{{MIC}_{\text{B} \text{i}\text{n} \text{c}\text{o}\text{m}\text{b}\text{i}\text{n}\text{a}\text{t}\text{i}\text{o}\text{n}}}{{MIC}_{\text{B} \text{a}\text{l}\text{o}\text{n}\text{e}}}$$

FICI ≤ 0.5 indicates synergistic effect, 0.5 < FICI ≤ 1 indicates additive effect, 1 < FICI ≤ 4 indicates indifference, and FICI > 4 indicates antagonistic effect.

### Biofilm detection by crystal violet staining

Crystal violet staining is a commonly used method for detecting and visualizing biofilms (Liu et al. 2023). Briefly, for biofilm inhibition, overnight cultured *S. aureus* was diluted with TSB to a final concentration of ~ 1 × 10^6^ CFU/mL. Equal volumes of the bacterial suspension and 2-fold serially diluted AKBA were added in a 96-well cell culture plates. After incubation at 37℃ for 24 h, the plates were washed to remove any loosely attached cells or debris. The left biofilms were fixed with formaldehyde, and then stained with 0.25% (wt/vol) crystal violet for 20 min. Excess crystal violet dye was washed by PBS, and 100 µL of ethanol was added to each well to destained the crystal violet. The absorbance at 570 nm (A_570nm_) was detected with a microplate reader (Bio-Rad, Hercules, USA) to quantify the biofilm biomass. As for biofilm eradication, the overnight cultured *S. aureus* was 1: 200 diluted with TSB, and added to a 96-well cell culture plates. After 24 h incubation at 37℃ the excessive planktonic cells were removed with PBS, and 200 µL of 2-fold serially diluted AKBA was added to each well. After incubation at 37℃ for 24 h, the plates were washed with PBS, and stained with crystal violet as described above.

### Biofilm detection by confocal laser scanning microscopy (CLSM)

The biofilms were cultured and treated with AKBA as described above in glass-bottomed cell culture dishes (NEST, Wuxi, China). After incubation at 37 °C for 24 h, the dishes were washed with PBS to remove unattached cells. Then, the dishes were stained with 500 µL PBS in the presence of 10 µM SYTO-9/PI (Thermo Fisher Scientific, Waltham, MA, USA) in the dark for 30 min. And the stained biofilms were observed by a CLSM (Zeiss LSM 800, Jena, Germany) with excitation/emission wavelengths of 488 nm/550 nm (SYTO9) and 540 nm/620 nm (PI), respectively (Zhang et al. 2023).

### Persister cells killing assay

The persister cells were cultured as described by Liu et al. (Liu et al. 2021). Briefly, *S. aureus* was prepared at 37 °C with shaking at 200 rpm for 24 h to platform stage. The bacterial suspension was washed three times with PBS, and adjusted to OD630 = 0.2 in centrifuge tubes. A 1 ~ 8×MIC of AKBA was added into each tube, and CFU counts were performed after incubation at 37℃ for 0, 2, 4, 6 and 8 h, respectively. Conventional antibiotics of VAN, CIP, DAP, and GEN (10×MIC) were used as controls.

### Resistance inducing assay

*S. aureus* was overnight cultured at 37℃ 180 rpm in TSB. And the bacterial suspension was further sub-cultured in fresh TSB to log-phased growth. The MIC was detected as mentioned above at the first day. After incubation at 37℃ for 24 h, the MIC was recorded, and the bacterial suspension in the wells of 0.5×MIC was 1000-fold diluted with MH broth, and further performed the MIC detection for the next day. Ciprofloxacin was used as a control during the test. The *S. aureus* was treated for a total of 20 days of serial passage in duplicate (Kim et al. 2018).

### RBC lysis activity determination

Human RBC was purchased from the Hemo Pharmaceutical and Biological Co (Shanghai, China). After centrifugation at 1000×rpm for 10 min, RBC was separated and further resuspended in PBS. Indicated concentrations of AKBA were added in equal volumes to RBC solution and incubated at 37 °C for 1 h. After centrifugation at 1000×g for 10 min, the absorbance at 540 nm (A_540_) was measured. Treatments with PBS and 1% TritonX-100 were used as negative and positive control, respectively (Farheen et al. 2023). The hemolysis rate was calculated as follows:

$$Hemolysis\,(\% ) = \left( {\frac{{{A_{sample}} - {A_{PBS}}}}{{{A_{TritonX - 100}} - {A_{PBS}}}}} \right) \times 100\%$$

### Cytotoxicity detected by CCK-8 kit

The cytotoxicity of AKBA was determined as previously reported by Liu et al. (Liu et al. 2020) with minor modifications. The cell lines of LO2, HepG2 and HSF were grown to logarithmic growth phase in Dulbecco’s modified Eagle’s medium (DMEM) or RPMI 1640 culture medium containing 10% fetal bovine serum at 37 °C 5% CO_2_. The cells were further inoculated into a 96-well plate at a density of 4 × 10^4^ cells per well and treated with 100 µL of DMEM medium with AKBA. The untreated cells were used as a control. After incubation at 37 °C 5% CO_2_ for 24 h, 10 µL of CCK-8 solution was added to each well. The absorbance at 450 nm (A_450_) was measured using microplate reader after further incubation for 1 h.

### Murine subcutaneous abscess model

Subcutaneous infection model of 7-week-old female ICR mice weighing 23–27 g was established and randomly divided into 3 groups: (1) Vehicle group, (2) AKBA (15 mg/kg) treatment group, and (3) vancomycin (VAN, 20 mg/kg) treatment group with 6 mice in each group. AKBA was prepared in a Cremophor EL/ethyl alcohol mixture (1:1, vol/vol) as the storage concentration. Overnight cultured MRSA ATCC 43,300 was washed and resuspended in saline to the concentration of 3 McF. Each mouse was injected s.c. with 0.2 mL of the bacterial suspension. At an hour post-infection, a single dose of the compounds was administered (s.c.). After 24 h post infection, the mice were euthanized, and the abscess area was calculated with calipers. And the infected skin was dissected and homogenized for bacterial load counting, H&E staining, and immunohistochemical (IL-1β, IL-6, and TNF-α), respectively. Meanwhile, to assess the in vivo toxicity by AKBA, the blood samples were collected from the orbital vein, and the level of organic function biomarkers were determined by Hitachi 7600 series automated biochemistry analyzer (Zhang et al. 2021).

### Statistical analysis

All experiments were performed independently in triplicate. The data were analyzed using Prism 8.0 (GraphPad Software, San Diego, CA, USA) and were expressed as the mean ± standard deviation (SD). Statistical significance was determined using unpaired Student’s t test or one-way ANOVA and Dunn’s multiple comparison test. A *P* value of < 0.05 was considered statistically significant.

## Results

### Bactericidal antimicrobial activity of AKBA against MRSA

**Fig. 1:**
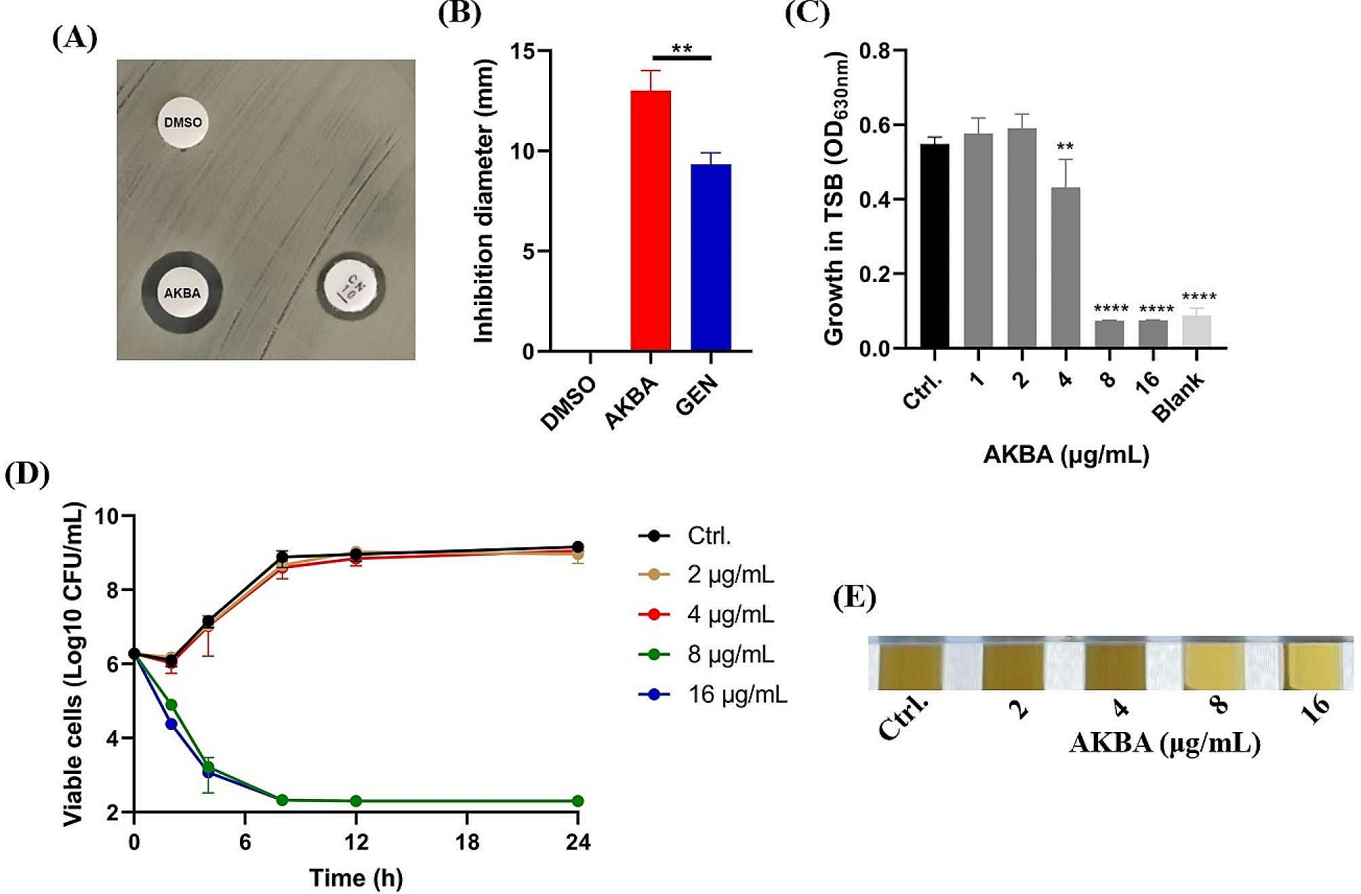
Bactericidal activity of AKBA against MRSA ATCC 43,300. **(A)** Antimicrobial susceptibility of AKBA determined by K-B test. AKBA: 6 µL. GEN: gentamycin, 10 µg. DMSO (6 µL) was used as a control. **(B)** Inhibition diameters quantification of the K-B test. **(C)** Growth inhibition activity by AKBA. **(D)** Time-killing curve of AKBA against ATCC 43,300. **(E)** Growth turbidity of ATCC 43,300 after treated with AKBA for 24 h

Firstly, the antimicrobial susceptibility of AKBA against the Gram-positive and Gram-negative pathogens were determined by micro-dilution assay. As shown in Table 1, AKBA exhibited effective antimicrobial activity against *S. aureus* (including MSSA and MRSA) with the MIC and MBC of 4–8 µg/mL and 8–16 µg/mL, respectively. Meanwhile, the *S. epidermidis* also showed the similar susceptibility to AKBA. However, no antimicrobial effect was observed by AKBA against Gram-negative pathogens or fungi with the MIC > 32 µg/mL. Further, the antimicrobial effect by AKBA against MRSA was further confirmed by the disc diffusion assay. As shown in Fig. 1A, MRSA ATCC 43,300 was resistant to gentamycin but susceptible to AKBA with the significantly increased inhibition zone (Fig. 1B). By growth inhibition assay, AKBA was found to significantly inhibit the MRSA ATCC 43,300 growth in TSB at the concentration of sub-MIC (4 µg/mL) (Fig. 1C), similarly, AKBA exhibited fully bactericidal activity against MRSA at the concentration of MIC (Fig. 1D). And the bactericidal effects could be last for 24 h (Fig. 1D and E).

**Table 1:** Antimicrobial susceptibility of AKBA against Gram-postive/negative pathogens

Strains	MIC (µg/mL)	MBC (µg/mL)
*S. aureus*		
ATCC 43,300^a^	8	8
ATCC 25,923	8	8
USA300 ^a^	4	16
MW2	8	8
*S. epidermidis*		
RP62A	8	16
ATCC 12,228	8	16
*E. coli*		
ATCC 25,922	> 32	> 32
*K. pneumoniae*		
ATCC 700,603	> 32	> 32
*A. baumannii*		
ATCC 19,606	> 32	> 32
*P. aeruginosa*		
PAO1	> 32	> 32
*E. faecalis*		
ATCC 29,212	> 32	> 32

a: MRSA

**Fig. 2:**
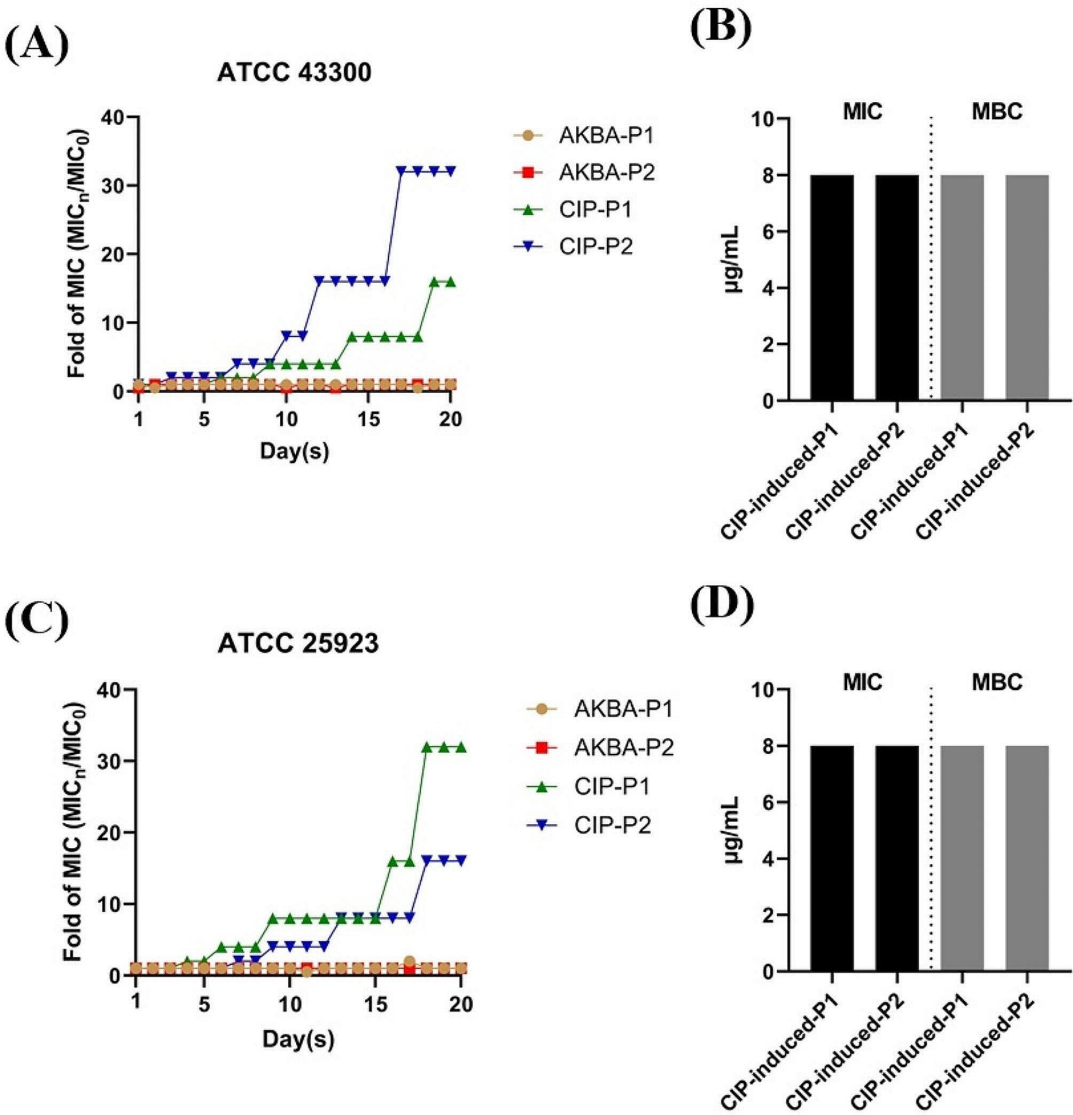
Low antimicrobial resistance inducing ability of AKBA. **(A)** Resistance inducing ability of AKBA against MRSA ATCC 43,300. **(B)** MIC determination of AKBA against CIP-induced resistant strains of ATCC 43,300. **(C)** Resistance inducing ability of AKBA against MSSA ATCC 25,923. **(D)** MIC determination of AKBA against CIP-induced resistant strains of ATCC 25,923. P1, parallel test (1) P2, parallel test (2) CIP was used as a positive control

In addition, no resistant mutation was occurred by MRSA ATCC 43,300 after 20 days of consecutive induction by sub-MIC of AKBA, although 16- to 32-fold of MIC increasement was observed by CIP treatment (Fig. 2A). And no cross-resistance was exhibited with the MIC and MBC of AKBA against CIP-induced resistant strains are still 8 µg/mL (Fig. 2B). Similarly, no resistant (Fig. 2C) or cross-resistant (Fig. 2D) by AKBA against MSSA ATCC 25,923 was exhibited.

**Fig. 3:**
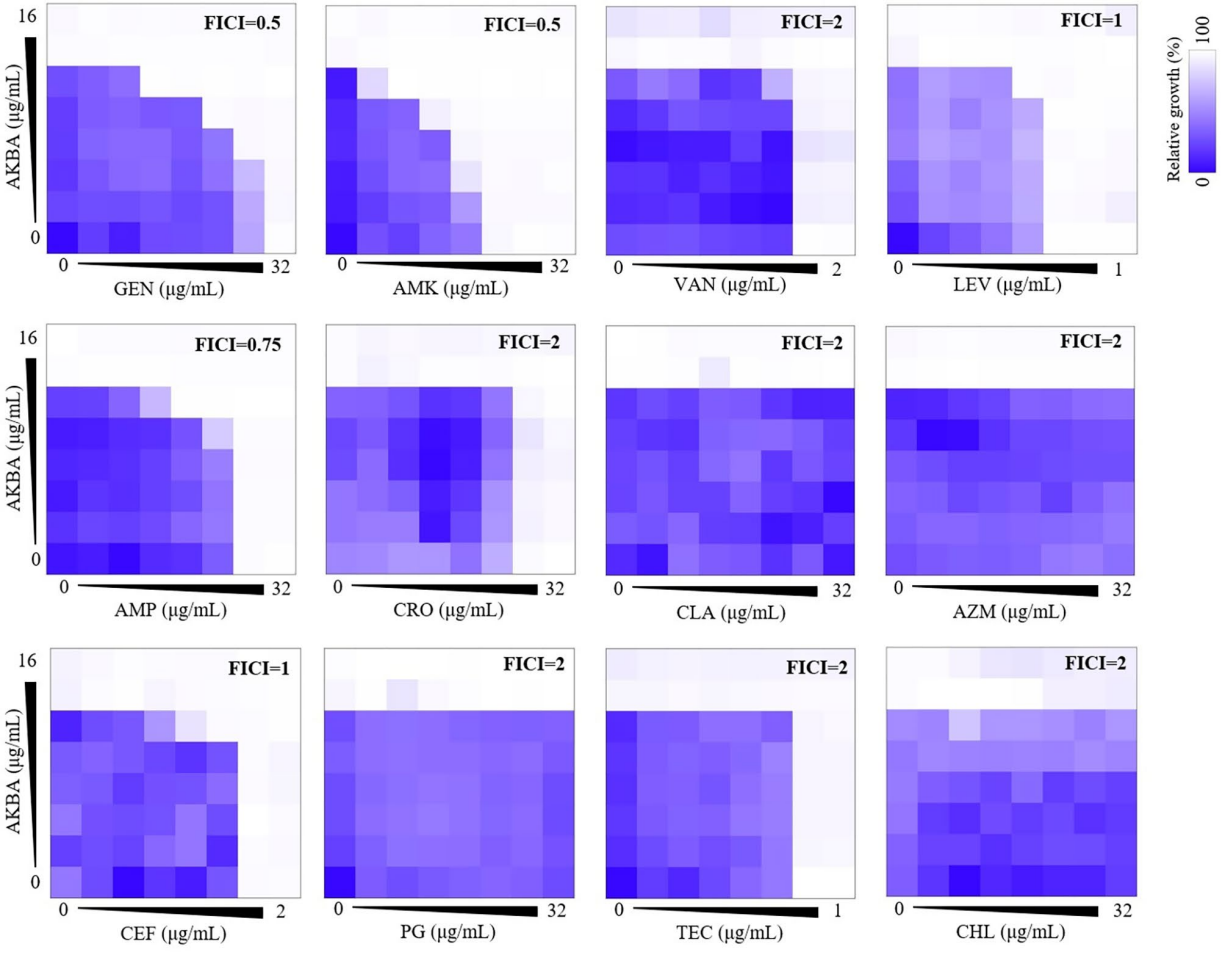
Drug combination between AKBA and conventional antibiotics determined by checkerboard assay. GEN, gentamycin. AMK, amikacin. VAN, vancomycin. LEV, levofloxacin. AMP, ampicillin. CRO, ceftriaxone sodium. CLA, clarithromycin. AZM, azithromycin. CEF, cefsulodine sodium. PG, penicillin G. TEC, teicoplanin. CHL, chloramphenicol

To intensify the clinical application and reduce the toxicity, drug combinational effects between AKBA and conventional antibiotics were explored. As shown in Fig. 3, AKBA exhibited synergistical antimicrobial activity with gentamycin (GEN) and amikacin (AMK) with the FICI of 0.5 both. However, no interaction was found between AKBA and other antibiotics with all the FICIs > 0.5. Further, we confirmed the synergy between AKBA with GEN (Table 2) or AMK (Table 3) in clinical isolates of MRSA and MSSA, respectively.

**Table 2:** Synergistic antimicrobial activity between AKBA and GEN against MRSA and MSSA clinical isolates

Strains	Resistant Pattern	Antimicrobial	MIC_alone_	Fold Change	MIC_in combination_	FICI	Outcome
SA2301	MRSA	AKBA	8	2	0.25	0.5	Synergy
		GEN	16	4	0.25		
SA2302	MRSA	AKBA	8	1	0.125	0.25	Synergy
		GEN	32	4	0.125		
SA2303	MRSA	AKBA	8	2	0.25	0.375	Synergy
		GEN	16	2	0.125		
SA2304	MRSA	AKBA	8	2	0.25	0.5	Synergy
		GEN	16	4	0.25		
SA2305	MRSA	AKBA	8	0.5	0.063	0.188	Synergy
		GEN	> 32	4	0.125		
SA2306	MRSA	AKBA	8	2	0.25	0.313	Synergy
		GEN	32	2	0.063		
SA2307	MRSA	AKBA	4	0.5	0.125	0.188	Synergy
		GEN	32	2	0.063		
SA2308	MRSA	AKBA	8	2	0.25	0.281	Synergy
		GEN	32	1	0.031		
SA2309	MRSA	AKBA	8	2	0.25	0.375	Synergy
		GEN	32	4	0.125		
SA2310	MRSA	AKBA	4	1	0.25	0.375	Synergy
		GEN	16	2	0.125		
SA23101	MSSA	AKBA	4	1	0.25	0.375	Synergy
		GEN	2	0.25	0.125		
SA23102	MSSA	AKBA	4	1	0.25	0.5	Synergy
		GEN	2	0.5	0.25		
SA23103	MSSA	AKBA	8	1	0.125	0.375	Synergy
		GEN	4	1	0.25		

**Table 3:** Synergistic antimicrobial activity between AKBA and AMK against MRSA and MSSA clinical isolates

Strains	Resistant Pattern	Antimicrobial	MIC_alone_	MIC_in combination_	Fold Change	FICI	Outcome
SA2301	MRSA	AKBA	8	2	0.25	0.5	Synergy
		AMK	4	1	0.25		
SA2302	MRSA	AKBA	8	2	0.25	0.5	Synergy
		AMK	8	2	0.25		
SA2303	MRSA	AKBA	8	1	0.125	0.375	Synergy
		AMK	2	0.5	0.25		
SA2304	MRSA	AKBA	8	2	0.25	0.5	Synergy
		AMK	4	1	0.25		
SA2305	MRSA	AKBA	8	1	0.125	0.375	Synergy
		AMK	0.5	0.125	0.25		
SA2306	MRSA	AKBA	8	2	0.25	0.375	Synergy
		AMK	8	1	0.125		
SA2307	MRSA	AKBA	4	1	0.25	0.5	Synergy
		AMK	8	2	0.25		
SA2308	MRSA	AKBA	8	1	0.125	0.25	Synergy
		AMK	2	0.25	0.125		
SA2309	MRSA	AKBA	8	2	0.25	0.5	Synergy
		AMK	4	1	0.25		
SA2310	MRSA	AKBA	4	1	0.25	0.5	Synergy
		AMK	8	2	0.25		
SA23101	MSSA	AKBA	4	0.5	0.125	0.375	Synergy
		AMK	1	0.25	0.25		
SA23102	MSSA	AKBA	4	1	0.25	0.375	Synergy
		AMK	2	0.25	0.125		
SA23103	MSSA	AKBA	8	2	0.25	0.5	Synergy
		AMK	2	0.5	0.25		

### Effective antibiofilm and anti-persister cells effects by AKBA

**Fig. 4:**
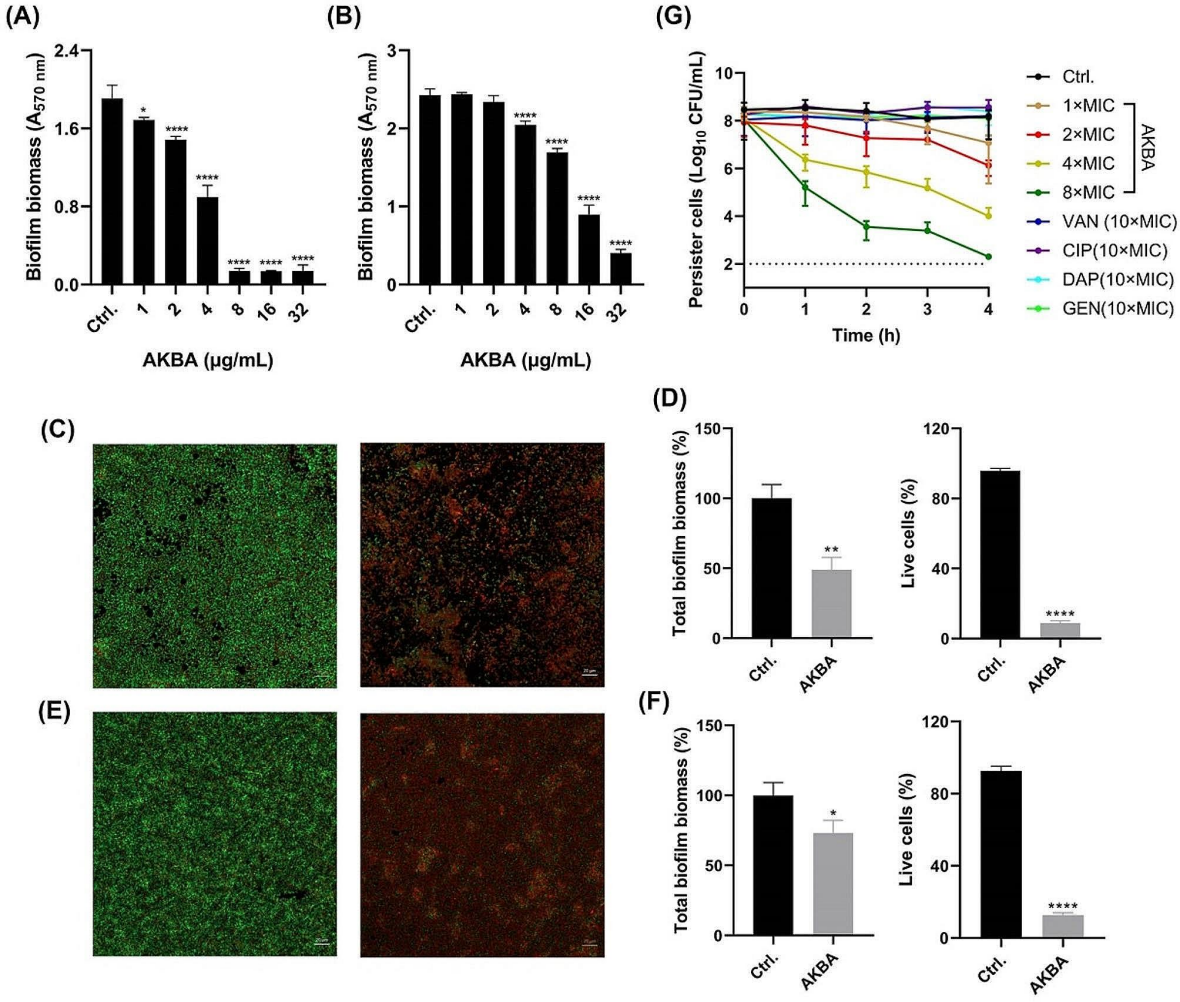
Antibiofilm effects of AKBA. **(A)** Biofilm inhibition activity of AKBA determined by crystal violet staining. **(B)** Biofilm eradication activity of AKBA determined by crystal violet staining. **(C)** Biofilm inhibition visualization by SYTO9/PI staining. **(D)** Fluorescence intensity quantitation of **(C)**. **(E)** Biofilm eradication visualization by SYTO9/PI staining. **(F)** Fluorescence intensity quantitation of **(E)**. The biofilm was stained with SYTO9/PI fluorescent probes. SYTO9 (green) and PI (red) were indicated viable cells and dead cells, respectively. **(G)** Persister killing activity of AKBA determined by time-killing assay. VAN/CIP/DAP/GEN at the concentration of ten-fold of MIC were used as controls

As widely reported, biofilms formed by MRSA showed high resistant to antimicrobials. However, in the present study, AKBA exhibited significant antibiofilm activity against MRSA. The crystal violet staining assay exhibited that AKBA could significantly inhibit the biofilm formation (Fig. 4A) and eradicated the preformed biofilms (Fig. 4B) at the concentration of 1 and 4 µg/mL, respectively, in a dose-dependent manner. The CLSM images by SYTO9/PI staining also confirmed the biofilm inhibitory (Fig. 4C and D) and eradication (Fig. 4E and F) effects by AKBA with reduced total biofilm biomass as well as the live cells in biofilms. Similarly, AKBA also showed effective bactericidal activity against MRSA persister cells at the concentration of 1-8x MIC in a dose- and time-dependent manner, although no antimicrobial effect was observed by 10× MIC of conventional antibiotics of VAN, CIP, daptomycin (DAP) and GEN (Fig. 4G).

### Low cytotoxicity of AKBA to mammal cells

**Fig. 5:**
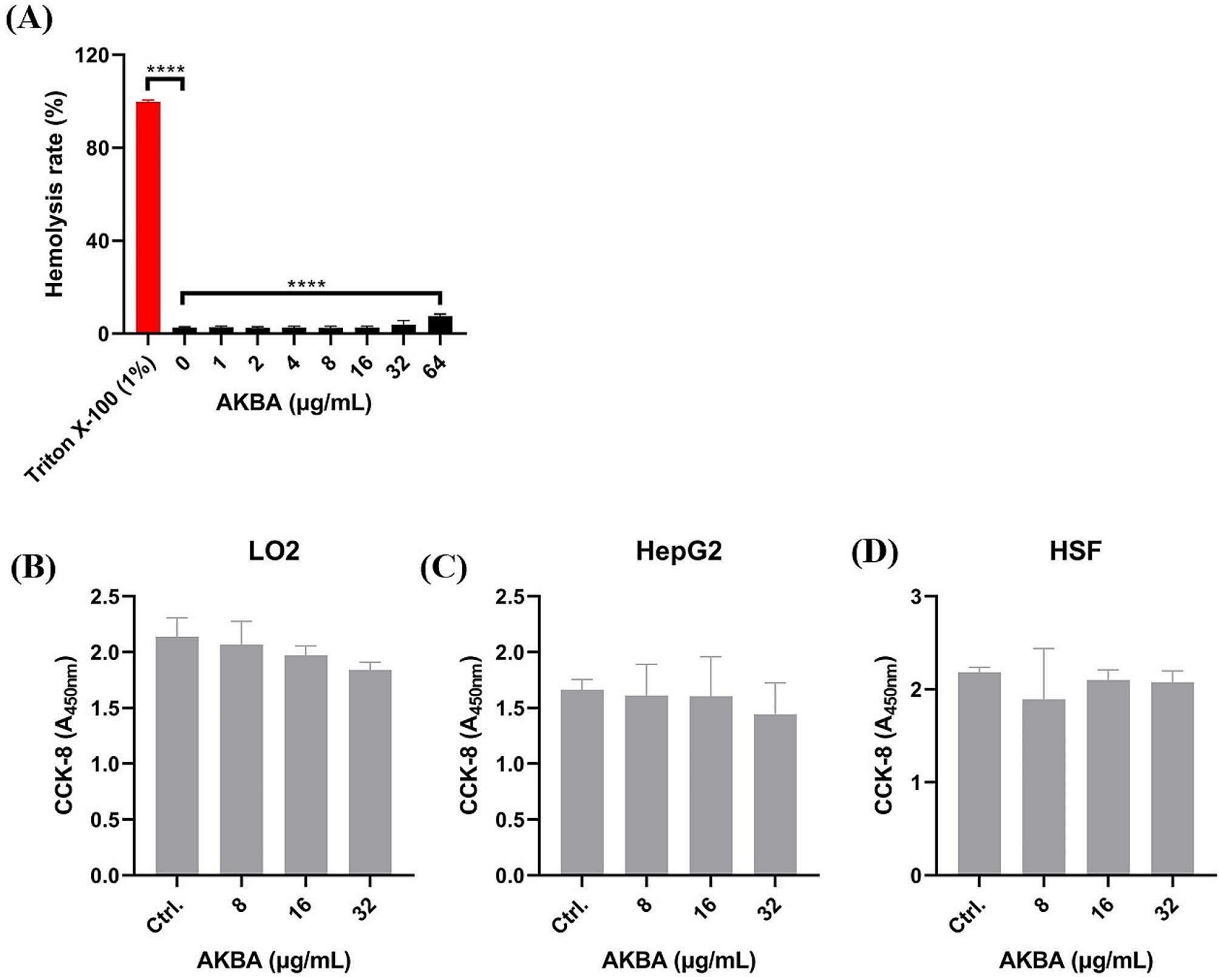
Cytotoxicity of AKBA. **(A)** Extremely low RBC lysis activity by AKBA. **(B-D)** Cytotoxicity detection of AKBA against cell lines of LO2 **(B)**, HepG2 **(C)** and HSF **(D)** by CCK-8 assay

Firstly, the human RBC hemolysis activity after AKBA treatment was detected. As we expected, AKBA showed no or only weak hemolysis even at the concentration up to 64 µg/mL (Fig. 5A). Further, the cytotoxicity of AKBA against mammal cells was determined. As shown in Fig. 5B and D, no significant cytotoxicity was observed in the cell lines of LO2 (Fig. 5B), HepG2 (Fig. 5C), and HSF (Fig. 5D). In all, AKBA is well-tolerant to human-derived cells with low hemolysis activity.

### In vivo antimicrobial activity and toxicity by AKBA

**Fig. 6:**
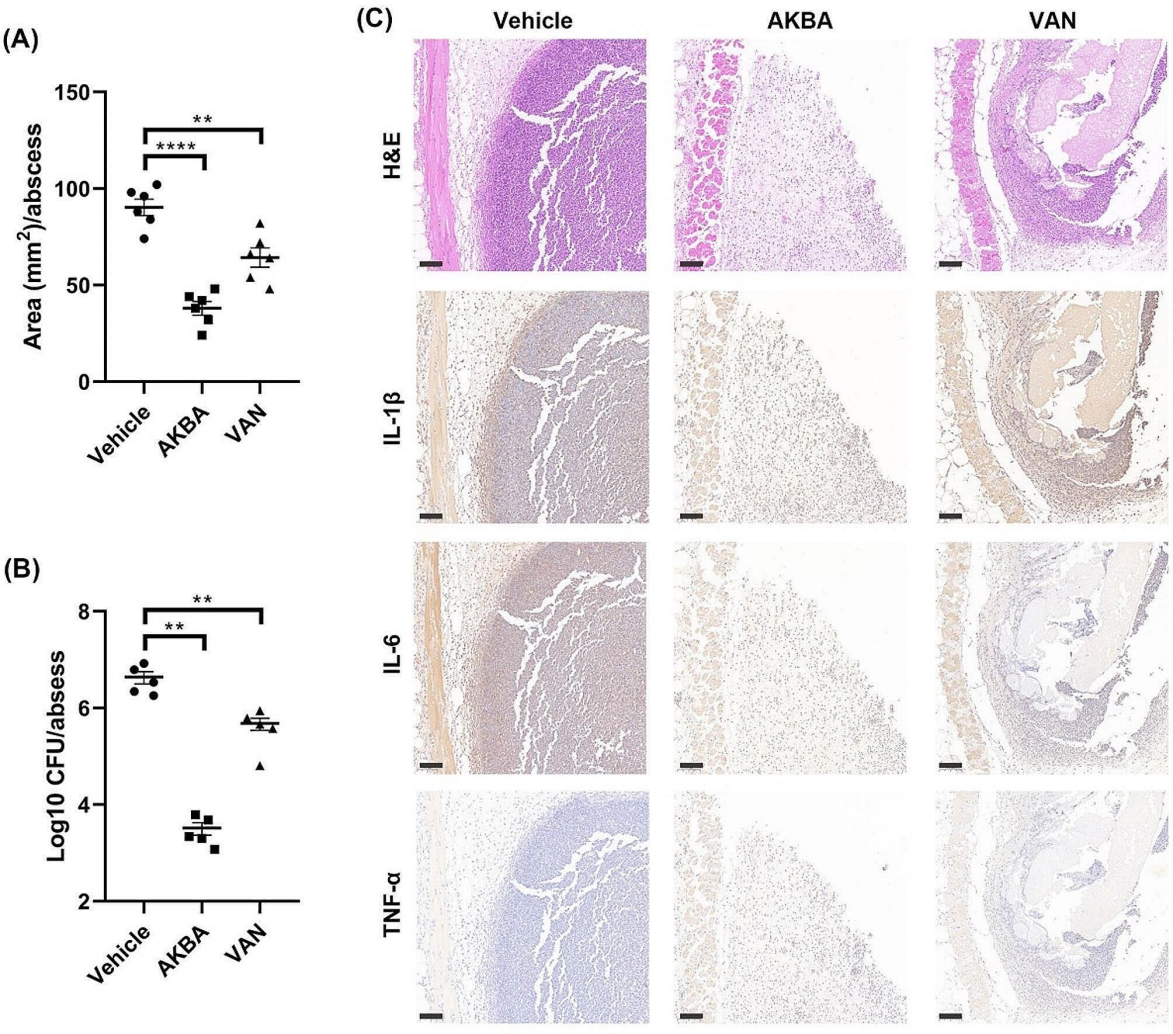
Antimicrobial effects of AKBA in an abscess infection model. Area size quantification **(A)** and viable cell counting **(B)** in abscess after treated with AKBA or VAN for 24 h. **(C)** Inflammatory cells infiltration and cytokines (IL-1β, IL-6, and TNF-α) expression observed by H&E staining and immunohistochemical, respectively

The antimicrobial efficacy of AKBA in vivo was detected in an abscess infection model. AKBA could significantly reduce the abscess area from (90.33 ± 10.39) to (38 ± 8.76) mm^2^ (Fig. 6A), and the viable cell counts was also significantly decreased from (6.57 ± 0.29) to (3.44 ± 0.29) Log10 CFU/mL after treatment with AKBA for 24 h, which even exhibited better antimicrobial activity than VAN (Fig. 6B). In consistence, By H&E staining observation, AKBA treatment largely reduced the inflammatory cells infiltration compared with the vehicle group or the VAN treated group. Similarly, the IHC images also showed more obvious reduction of inflammatory cytokines (IL-1β, IL-6 and TNF-α) in the AKBA treated group than the VAN treated group (Fig. 6C).

Meanwhile, the in vivo toxicity by AKBA was also assessed. As shown in Figure S1A, the mice were well tolerant to 20–40 mg/kg of AKBA treatment. Thus, 40 mg/kg of AKBA by intraperitoneal injection was used to further assess the in vivo toxicity to organs. As we expected, the levels of the liver function biomarker glutamic-pyruvic transaminase (ALT, Figure S1B), kidney function biomarker urea nitrogen (BUN, Figure S1C), and myocardium function biomarker creatine kinase (CK, Figure S1D) exhibited no statistical difference between the vehicle and AKBA treated group.

## Discussion

The increased antibiotic resistance of MRSA and its ability to form biofilms bring great challenges to clinical treatment. New antibacterial development has become an urgent need. The present study found that the Nrf2 activator AKBA showed effective antibacterial activity against MRSA and its biofilms with low probability of resistance reduction. Meanwhile, synergistical antimicrobial activity between AKBA and GEN or AMK was also observed. In vivo toxicity and antimicrobial efficacy were also assessed in an abscess infection model. The strong antibacterial activity and low cytotoxicity of AKBA makes it potentially to be an alternative for the treatment of refractory infections caused by *S. aureus*.

Drug repurposing has many advantages that cannot be replaced by the development of antibiotics in traditional ways. For example, lower overall development costs, shorter development time, and extremely low toxicity risks (Zheng et al. 2018). In this study, AKBA exhibited antibacterial activity against MRSA with MICs of 4–8 µg/mL. And *S. epidermidis* also showed the same susceptibility to AKBA. In recent years, researchers have discovered some highly promising antibacterial drugs against *S. aureus* through drug repurposing. For example, retinol like anti-tumor drugs CD437 and CD1530 exhibit significant anti-*S. aureus* activity at microgram per milliliter level. However, CD437 and CD1530 showed significant cytotoxicity on liver tumor cell HepG2 at the concentration of 3–5 µg/mL. And when its concentration reaches 20 µg/mL, there is also significant cytotoxicity to normal liver cell LO2 (Kim et al. 2018; Tan et al. 2019). Therefore, as repurposed antimicrobials, AKBA is safer than CD437 and CD1530. In addition, in recent years, antibacterial drugs such as auranofin (Sharma et al. 2021), penfluridol (Liu et al. 2021a), and idarubicin (She et al. 2020), which have been newly discovered through drug repurposing. But these drugs are still stagnated in the experimental stage due to some factors such as cytotoxicity, in vivo metabolic defects, or stability. However, AKBA not only has strong antibacterial activity, but also has extremely low cytotoxicity and great potential as an antimicrobial.

*S. aureus* has a strong ability to adapt to various adverse conditions and to develop resistance to almost all antibiotics. The resistance produced by *S. aureus* usually poses a serious health burden to the host. These drug resistances are often generated through mutations and rearrangements in the bacterial genome, or through horizontal transmission of drug resistant genes (Pulingam et al. 2022). On the other hand, the emergence of antibiotic resistance phenotype may also be due to the combined effects of the genetic background of the strain and certain environmental factors. For example, indirect regulation of drug resistance can be achieved by altering the expression of resistant genes or the specificity of substrate binding (Pope et al. 1998). In addition, certain insertion elements can alter antibiotic resistance by turning on or off the expression of related genes (Fujimura et al. 2008). In clinical practice, the resistance of *S. aureus* is usually established through multiple steps. In this study, we found that AKBA with sub-MIC did not induce the development of drug resistance in *S. aureus* even after 20 days of passage. Therefore, AKBA is extremely difficult to induce bacterial resistance, and has the advantages that conventional antibiotics cannot replace.

AKBA exhibited significant antibiofilm effects against *S. aureus*. The process of biofilm lifecycle mainly includes initial adhesion, initial biofilm formation, maturation, and dispersion (Liu et al. 2020). The formation of *S. aureus* biofilm is mainly regulated by the *agr*-related quorum sensing system. The quorum sensing system can generate self-induced signal peptides. As bacteria gathered, the concentration of the signaling molecules increases, and when exceeded the threshold, the expression of biofilm related genes (such as *icaA/B/C/D* operon) can be induced. Which will promote the production of extracellular polysaccharides and other biofilm matrix components (Boles et al. 2008; Zhang et al. 2018). Therefore, the Agr system is a key factor in regulating the formation of *S. aureus* biofilm. In our study, we found that 4 µg/mL of AKBA can not only inhibit the biofilm formation, but also significantly eradicate the pre-formed biofilm against *S. aureus*. Therefore, we speculate that the anti-biofilm mechanism of AKBA could be mediated by its interference with the Agr system. However, further study is needed in the future.

AKBA has strong antibacterial activity against MRSA and its clinical strains with MICs of 4–8 µg/mL. AKBA can also inhibit the biofilm formation of MRSA and effectively remove the pre-formed biofilm. AKBA hardly induces the resistance formation of *S. aureus*, and its cytotoxicity is extremely low. AKBA has great potential to development as an alternative treatment for refractory MRSA related infections.

### Electronic supplementary material

Below is the link to the electronic supplementary material.

Supplementary Material 1: Fig. S1 In vivo toxicity by AKBA. (A) Time-survival curve of mice after treated with a single dose of AKBA. Effects of AKBA on the serum level of the biomarkers of ALT (B), BUN (C), and CK (D), respectively

## Data Availability

Date will be made available on request.
