# Supplementary figures and images for: Drug repurposing: insights into the antimicrobial effects of AKBA against MRSA
Source: AMB Express. 2024 Jan 6;14:5. doi: 10.1186/s13568-024-01660-0 (PMC10771487; doi:10.1186/s13568-024-01660-0)

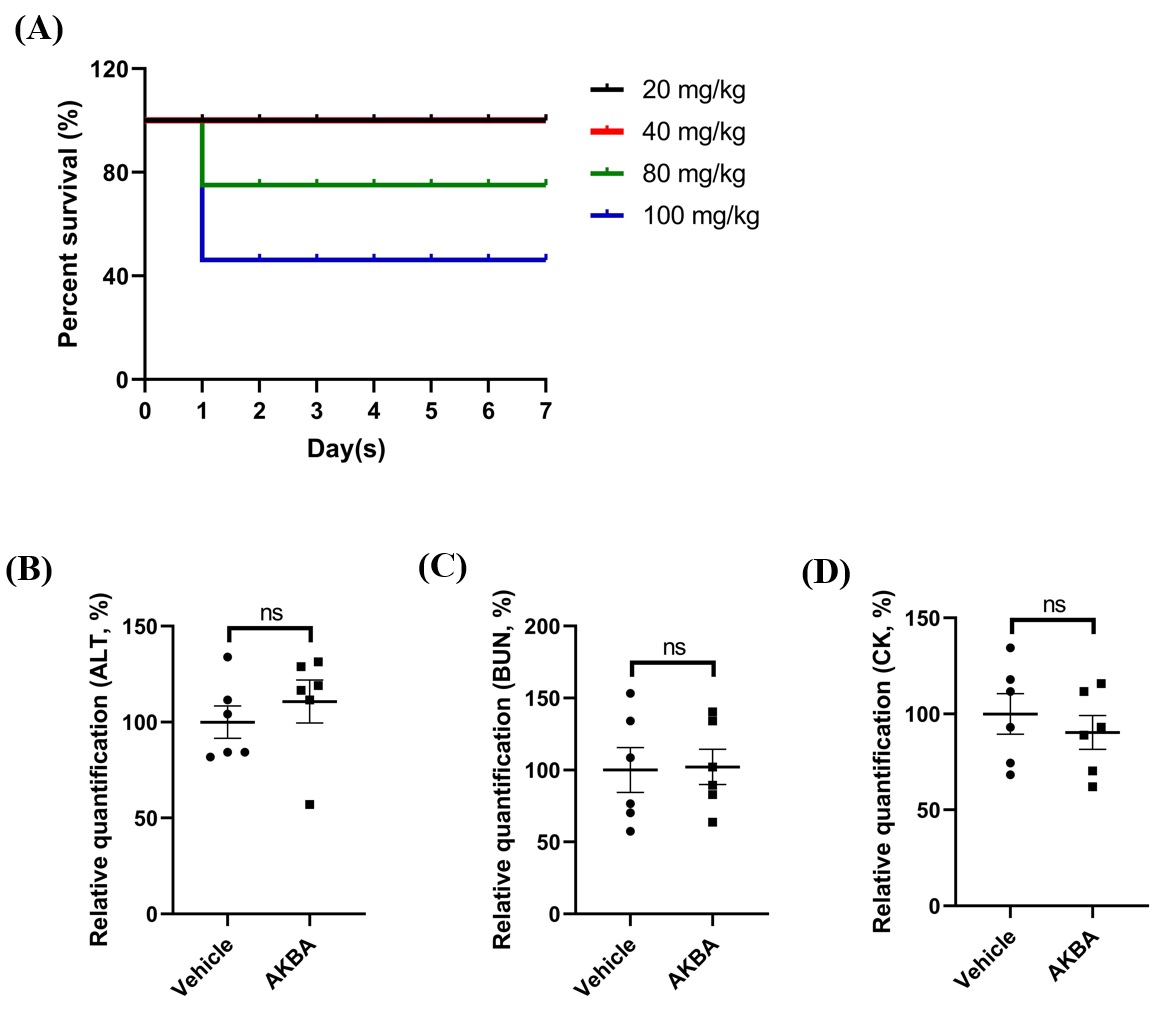

Supplement: Supplementary file 1 — Supplementary Material 1: Fig. S1 In vivo toxicity by AKBA. (A) Time-survival curve of mice after treated with a single dose of AKBA. Effects of AKBA on the serum level of the biomarkers of ALT (B), BUN (C), and CK (D), respectively [file 13568_2024_1660_MOESM1_ESM.jpg]
